# 109. Develop and Implement a Novel Pediatric Antimicrobial Stewardship Program in a Non-Freestanding Children’s Hospital Located in an Adult-Centered Community Hospital in San Joaquin Valley, California

**DOI:** 10.1093/ofid/ofab466.311

**Published:** 2021-12-04

**Authors:** Tsung-Chi Lien, Laurie Covarrubias, Alice Ip, Harlan Husted, Emi Suzuki, Chokechai Rongkavilit

**Affiliations:** 1 Community Regional Medical Center, Fresno, California; 2 University of California San Francisco, Fresno Branch Campus, Fresno, California

## Abstract

**Background:**

A pediatric-specific antimicrobial stewardship program (Ped ASP) has been shown to optimize antimicrobial use, improve patient outcomes, and reduce healthcare expenditures in this population. Opportunities and challenges exist when developing a Ped ASP for a children’s hospital within an adult-centered medical center primarily due to mixed infrastructure. The objective of this study is to provide process and outcome data of a new Ped ASP in a non-freestanding children’s hospital within an adult-centered tertiary hospital.

**Methods:**

A pediatric infectious disease physician and four pediatric pharmacists designed a Ped ASP utilizing direct and indirect patient care activities to optimize pediatric antimicrobial use in 21 bed-pediatric services within a 685-bed, adult-centered medical center. Implemented in 2020, Ped ASP activities include thrice weekly chart reviews followed by handshake rounds and quarterly reviews of documented interventions. The Ped ASP team also developed policies, education, and other resources to further guide appropriate antimicrobial use, in collaboration with the adult team.

**Results:**

Ped ASP was initiated on general pediatric (PED) and pediatric intensive care (PICU) units. In 2020, a total of 286 charts were reviewed with 199 antibiotic interventions provided, including optimization of antimicrobial selection (23%), IV-to-PO conversion (15%), and antimicrobial dosage adjustment (13%). Annual average antibiotic length and days of therapy per 1000 patient-days were 241 and 290 respectively in PED, and 388 and 432 in PICU. The overall trend from 2020 to 2021 decreased in PED but increased in PICU (Fig. 1). The ratio of narrow to broad spectrum antibiotic use increased for both PED and PICU (Fig. 2). Simultaneously, a pediatric-specific antibiogram, extended-infusion protocol of beta-lactams, and neonatal sepsis treatment algorithm were developed and implemented.

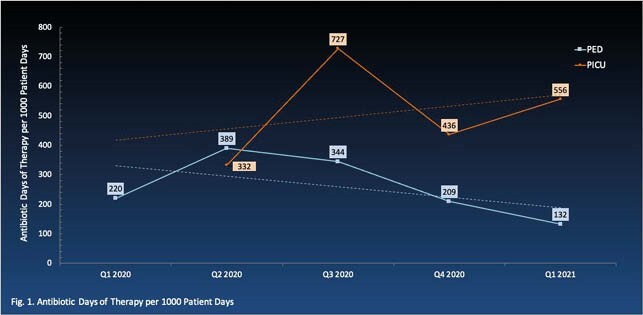

Antibiotic Days of Therapy per 1000 Patient Days

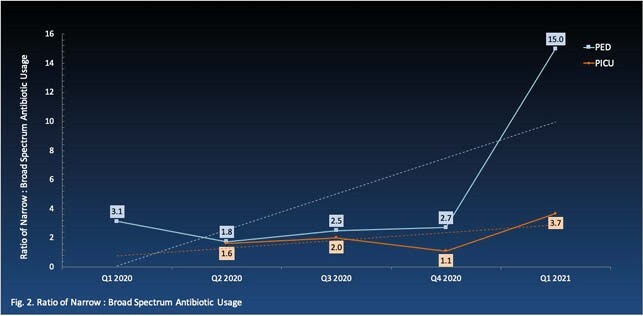

Ratio of Narrow: Broad Spectrum Antibiotic Usage

**Conclusion:**

A Ped ASP was successfully developed in a non-freestanding children’s hospital. Continual metrics served as an important tool to identify areas for improvement. Future goals include expansion of Ped ASP to other service lines, enhanced ASP education and development of additional pediatric antimicrobial treatment pathways.

**Disclosures:**

**All Authors**: No reported disclosures

